# Knockdown of *PagSAP11* Confers Drought Resistance and Promotes Lateral Shoot Growth in Hybrid Poplar (*Populus alba* × *Populus tremula* var. *glandulosa*)

**DOI:** 10.3389/fpls.2022.925744

**Published:** 2022-06-24

**Authors:** Su Jin Park, Eun-Kyung Bae, Hyunmo Choi, Seo-Kyung Yoon, Hyun-A Jang, Young-Im Choi, Hyoshin Lee

**Affiliations:** ^1^Department of Forest Bioresources, National Institute of Forest Science, Suwon, South Korea; ^2^Forest Biomaterials Research Center, National Institute of Forest Science, Jinju, South Korea; ^3^Department of Forest Sciences, Seoul National University, Seoul, South Korea; ^4^National Forest Seed and Variety Center, Forest Service, Chungju, South Korea

**Keywords:** stress-associated protein, *PagSAP11*, drought tolerance, lateral shoot branching, bud dormancy, hybrid poplar

## Abstract

Plants have evolved defense mechanisms to overcome unfavorable climatic conditions. The growth and development of plants are regulated in response to environmental stress. In this study, we investigated the molecular and physiological characteristics of a novel gene *PagSAP11* in hybrid poplar (*Populus alba* × *Populus tremula* var. *glandulosa*) under drought stress. *PagSAP11,* a stress-associated protein (SAP) family gene, encodes a putative protein containing an A20 and AN1 zinc-finger domain at its N- and C-termini, respectively. Knockdown of *PagSAP11* transgenic poplars (*SAP11*-Ri) enhanced their tolerance to drought stress compared with wild type plants. Moreover, the RNAi lines showed increased branching of lateral shoots that led to a gain in fresh weight, even when grown in the living modified organism (LMO) field. In *SAP11*-Ri transgenic plants, the expression levels of genes involved in axillary bud outgrowth and cell proliferation such as *DML10*, *CYP707A* and *RAX* were increased while the *DRM* gene which involved in bud dormancy was down-regulated. Taken together, these results indicate that *PagSAP11* represents a promising candidate gene for engineering trees with improved stress tolerance and growth during unfavorable conditions.

## Introduction

Plants lead a sedentary life; therefore, they have developed various mechanisms to adapt to changes in the surrounding environment (El-Sappah et al., 2022). Drought is an adverse environmental condition that can affect plant growth and development (El-Sappah and Rather, 2022). Global changes in drought have the potential to trigger widespread die-offs of trees, which could further exacerbate global warming and thus affect ecosystem products and service globally. Forest trees have adapted various defense strategies for drought stress (Lata and Prasad, 2011; Jia et al., 2020; Kim et al., 2020; Jian et al., 2021). For example, plants attempt to minimize water loss by reducing transpiration rate and surface area of the leaves (Estravis-Barcala et al., 2020). At the molecular level, dehydration-responsive-element-binding (DREBs) proteins, WRKY transcription factor and NAC (NAM, ATAF, and CUC) transcription factor regulate the expressions of stress-responsive genes such as *LEA* (late-embryogenesis) and aquaporin genes (Harfouche et al., 2014; Estravis-Barcala et al., 2020).

One such important family of stress response regulatory proteins, the Stress-Associated Protein (SAP) genes encode a novel class of zinc-finger proteins that are well conserved among various plant species. A distinct feature of SAP protein is the presence of A20/AN1 zinc-finger domains. The A20 zinc finger domain has Cx_2-4_Cx_11_Cx_2_C consensus sequence at the N-terminus whereas AN1 zinc finger domain has two consensus sequences: Cx_2_Cx_9-12_Cx_1-2_Cx_4_Cx_2_Hx_5_HxC and the Cx_4_Cx_9-12_Cx_1-2_Cx_4_Cx_2_Hx_5_HxC at the C-terminus (Jin et al., 2007). The A20 domain of SAPs was first identified as a tumor necrosis factor (TNF) induced gene from the human umbilical cord (Opipari et al., 1990). Furthermore, the AN1 domain was first identified in *Xenopus laevis*, a protein encoded by animal hemisphere 1 (AN1) maternal RNA (Jin et al., 2007). SAP family genes have been isolated from various plants, including *Arabidopsis thaliana*, *Populus euphratica*, *Populus trichocarpa*, *Medicago truncaula*, *Brassica napus* and *Zea mays* after the identification of the first *SAP* gene (*OsSAP1*) in *Oryza sativa* (Vij and Tyagi, 2006; Jin et al., 2007; Jia et al., 2016).

To date, several *SAP* genes have been identified that play important roles in the regulation of plant responses to abiotic stress. *OsSAP1* improves tolerance to cold, drought, and salinity stresses in transgenic tobacco (Mukhopadhyay et al., 2004). Overexpression of *AtSAP5* confers salt, drought and osmotic tolerance in *Arabidopsis* (Kang et al., 2011). While, *GmSAP16*-overexpressing plants showed improved resistance to drought and salinity stress and increased abscisic acid (ABA) sensitivity in *Arabidopsis* (Zhang et al., 2019). Further, overexpression of *MdSAP15* in *Arabidopsis* led to enhanced osmotic and drought stress tolerance (Dong et al., 2018). In addition, silencing of *SlSAP4* resulted in reduced resistance to *Botrytis cinerea* infection in tomato (Liu et al., 2019). In woody plants, it was identified that down-regulation of *PagSAP1* enhances tolerance to salt stress in hybrid poplar (Yoon et al., 2018). Li et al. (2019) also reported that overexpression of *PtSAP13* increased resistance to salt stress in *Arabidopsis*. Although several *SAPs* have been identified in various plants, their exact biological functions in woody plants are not yet fully understood.

Owing to its rapid growth rate, high productivity and extensive adaptation to environment, poplar is used as resource for biofuels and other bioproducts. With the availability of its full genome sequence and the establishment of transgenic techniques, poplar has become a model organism for studying woody plants (Bradshaw et al., 2001; Sannigrahi et al., 2010; Guo et al., 2012). Considering that productivity of poplars closely depends on water availability (Khan et al., 2016), increased drought tolerance is important factor for improving its utility.

In this study, we carried out the genetic and molecular characterization of *PagSAP11* in hybrid poplar (*Populus alba* × *Populus tremula* var. *glandulosa*). We generated *PagSAP11* transgenic poplar plants to investigate the function of *PagSAP11*. Downregulated expression of *PagSAP11* in transgenic plants results in increased number of branches and leads to improved tolerance to drought stress. Our results suggest that *PagSAP11* plays an important role in drought stress response and regulation of plant architecture.

## Materials and Methods

### Plant Materials and Growth Conditions

Hybrid poplars [*P. alba* × *P. tremula* var. *glandulosa*, clone Bonghwa (BH)] were used as both WT and transgenic plants in this study. Control and transgenic plants were propagated *in vitro* prior to acclimation in pots containing commercial horticultural potting soil. The plants were then cultivated in growth chambers (16 h light/8 h dark cycle; light intensity, 150 μM m^−2^ s^−1^; 24°C), followed by acclimation in soil and growth for an additional 6 weeks until they reached a plant height of approximately 15 cm. Then, plants were transferred to LMO field and grown there at the Forest Bioresources Department of the National Institute of Forest Science, Republic of Korea (latitude 37.2 N, longitude 126.9E).

For tissue-specific gene expression analysis, leaf, stem, and root tissues were collected from 1-year-old poplar plants whereas flower tissues were collected from 25-year-old hybrid poplar plants.

### Isolation and Characterization of *PagSAP11*

A cDNA library was constructed as described by Lee et al. (2005) and blasted against previously identified plant SAP genes. The selected cDNA clone (GenBank accession number CX653572.1) was sequenced, and the deduced amino acid sequences and molecular weights of the products were determined using CLC Main Workbench (Qiagen, Aarhus, Denmark). Multiple sequence alignment and phylogenetic tree analysis were performed using the ClustalW (Sievers et al., 2011) and MEGA11 (Tamura et al., 2021) based on previously deduced amino acid sequences of SAP proteins such as from AtSAP3 (NP_001189620.1), AtSAP5 (NP_566429.1), AtSAP7 (NP_192941.1), AtSAP9 (NP_194013.1), and AtSAP10 (NP_194268.1) from *A. thaliana*; GmSAP5 (XP_003540534.1) from *Glycine max*; MeSAP5 (XP_021624685.1) from *Manihot esculenta*; OsSAP1 (XP_015651267), OsSAP11 (XP_015651039.1), and OsSAP15 (XP_015639532.1) from *O. sativa*; PeSAP5 (XP_011007335.1) from *Populus euphratica*; PtSAP4 (XP_002298442.1) and PtSAP11 (XP_002314027.1) from *P. trichocarpa*; PpSAP5 (XP_007218519.1) from *Prunus persica*; RcSAP5 (XP_002513177.1) from *Ricinus communis*; VvSAP5 (XP_003633555.1) from *Vitis vinifera*.

### RNA Isolation and Reverse-Transcription PCR Analysis

Total RNA was isolated from various tissues of hybrid poplar (clone BH) plants using RNeasy Plant Mini Kit (Qiagen, Hilden, Germany) according to manufacturer’s instructions. Reverse-transcription quantitative PCR (RT-qPCR) primers were designed using the Primer3 program.^1^ Gene expression levels were determined using the 2^−△△Ct^ method (Livak and Schmittgen, 2001). The first-strand cDNA was synthesized using EcoDry^™^ premix (Takara, Kyoto, Japan) and RT-qPCR was performed using the SYBR Green PCR Master Mix (Bio-Rad Laboratories, CA, United States) and a CFX96^™^ Real-Time System (Bio-Rad Laboratories, CA, United States). The cycling conditions for amplification were performed according to the manufacturer’s instructions. All primers used for RT-qPCR are listed in Supplementary Table 1.

### Subcellular Localization of *PagSAP11*

To determine the subcellular localization of *PagSAP11*, eGFP was fused in-frame with the C-terminus of *PagSAP11*. The coding sequence of *PagSAP11* was amplified from the genomic DNA of hybrid poplar (clone BH) using gene-specific primers (Supplementary Table 1). The PCR products were cloned into the pENTR/dTOPO Vector (Invitrogen, CA, United States). Correct construction of the clones was confirmed by sequencing. The *PagSAP11* fragment was then cloned into the Gateway version of pCsVMV-eGFP-N-999 vector by LR recombination reaction (Kim et al., 2008). Next, CsVMV::PagSAP11-eGFP recombinant plasmid was introduced into poplar protoplasts using the polyethylene glycol method (Abe and Theologis, 1998). The localization of PagSAP11-eGFP was observed using confocal laser scanning microscope (Leica, TCS SP5, Wetzlar, Germany).

### Generation of Transgenic Poplars Harboring OX and RNAi Vectors

**Figure 1:**
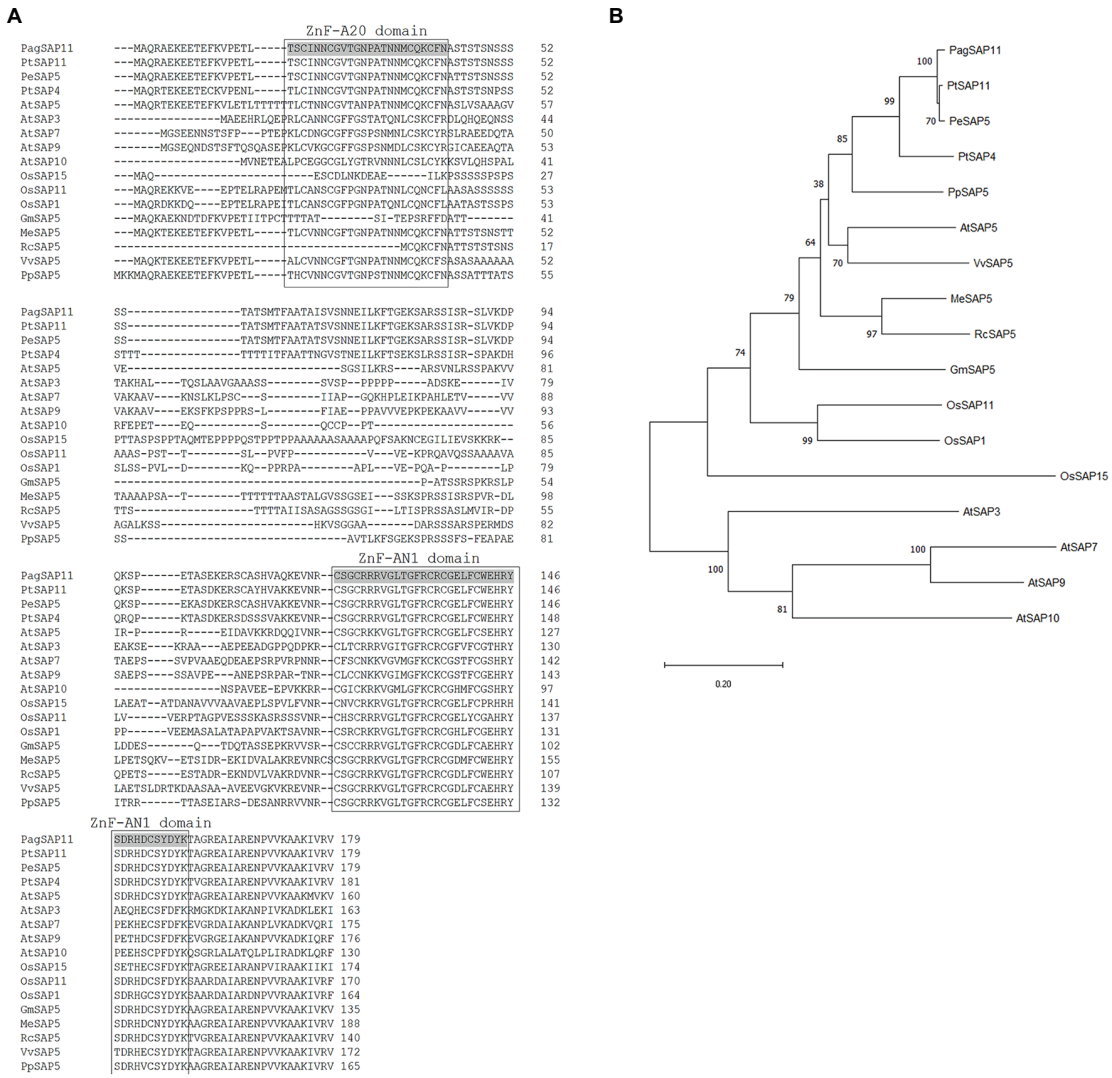
Comparison of the predicted protein sequences of *PagSAP11* with other plant SAP proteins. **(A)** Multiple sequence alignment of *PagSAP11* and related proteins from *Populus trichocarpa*, *Populus euphratica*, *Arabidopsis thaliana*, *Oryza sativa*, *Glycine max*, *Manihot esculenta*, *Ricinus communis*, *Vitis vinifera,* and *Prunus persica*. **(B)** Phylogenetic analysis of proteins from **(A)**.

PCR amplification of *PagSAP11* was performed using a specific primer set, using a forward primer (5′-GAA TCT AGA GGA TCC ACT TTT GAG AAG ATG-3′) and a reverse primer (5′-ATA AAC AAT GAG CTC AGT CCA TGG C-3′). The cycling conditions for amplification were performed according to the manufacturer’s instructions. To construct the RNAi vector, a partial sequence of *PagSAP11* was inserted into the pH7GWIWG vector (Karimi et al., 2002) using the Gateway system (Invitrogen, CA, United States). A schematic diagram of vector construction is shown in Figure 1. To construct the vector for *PagSAP11*-OX, the PCR products were ligated into the pGEM-T Easy Vector (Promega, WI, United States). The vector was digested with *Xba*I and *Sac*I and inserted into the pBI121 binary vector. Plant transformation was performed as previously described by Choi et al. (2005). The transformed cells were selected on MS medium containing 1 mg/l 2, 4-D, 0.1 mg/l naphthaleneacetic acid (NAA), 0.01 mg/l benzyladenine (BA), and 2 mg/l hygromycin for the RNAi lines, or 50 mg/l kanamycin for the OX lines. Putative transgenic shoots were regenerated in woody plant medium (WPM; Duchefa, Haarlem, Netherlands) containing 1.0 mg/l *t-*zeatin, 0.1 mg/l BA, and 0.01 mg/l NAA under the same concentrations of antibiotics. The regenerated shoots were transferred to MS medium containing 0.2 mg/l indole-3-butyric acid (IBA) for rooting.

### Stress Treatment

To estimate expression levels of *PagSAP11* under stress conditions, WT poplar (clone BH) plants were grown in soil pots under the normal watering conditions for 8 weeks and then subjected to the following treatments: water (control), 150 mM NaCl and 10% PEG for 24 h. For heat and cold treatments, 2-month-old WT poplars grown in soil under normal conditions were exposed to 40°C or 4°C for 24 h in growth chambers (Vision Scientific, Daejeon, Korea). To investigate response to drought stress, WT and transgenic poplar trees were grown in soil under the normal condition for 8 weeks and then watering was suspended for 10 days. Afterward, the photochemical efficiency of photosystem II (PSII) and chlorophyll contents were measured in the 6-8^th^ leaves from the top using a plant efficiency analyzer (Hansatech Instruments, Morfolk, UK) and ethanol extraction method (Lee et al., 2016; Cho et al., 2021), respectively. To analyze water loss, the detached leaves exposed to dehydration on a clean-bench (Hu et al., 2010). Briefly, five fully expanded leaves from three 8-week-old soil-acclimated WT and transgenic poplar trees were detached and placed on a clean-bench. Leaves were weighed at the indicated times to measure the rate of water loss.

### Statistical Analysis

All data represent the mean values (±standard deviation) from at least three independent replicates. Statistical analysis was conducted using the Student T-test, and significance level is indicated by asterisks.

## Results

### Isolation and Gene Characterization of *PagSAP11*

Analysis of the expression profiles under drought stress will provide insights for investigation of candidates; thus, we first analyzed the expression patterns of *SAP* family genes of the hybrid poplar (*P. alba* × *P. tremula* var. *glandulosa*, clone BH) in response to drought stress using qRT-PCR. We detected *SAP* genes based on the public database of *P. trichocarpa*.^2^ As a result, except for three *SAP*s (*SAP3*, *−14* and *− 18*), the expression of other *SAPs* increased under drought conditions (Supplementary Figure S1). Among them, four *SAPs* (*SAP7*, *−11*, *−13* and *− 16*) exhibited significantly up-regulated expression patterns under drought stress. Notably, according to the previous report, the expression of *PtSAP11* increased under salt treatment (Li et al., 2019). Taken together, we selected *SAP11* for further investigation of gene function.

**Figure 2:**
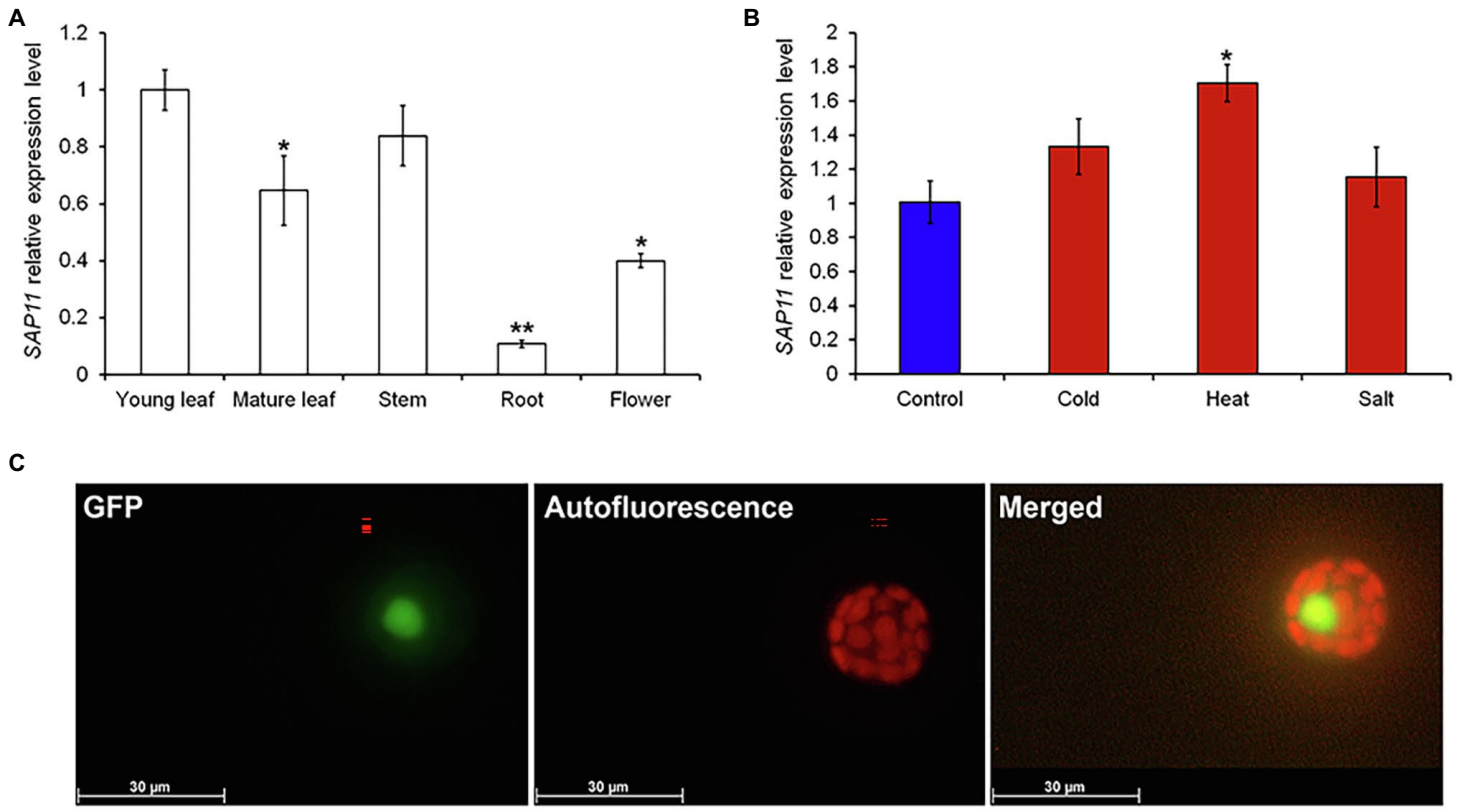
Characterization of *PagSAP11*. **(A)** Expression analysis of *PagSAP11* gene in poplar tissues by qRT-PCR: young leaf, mature leaf, stem, root and flower. **(B)** Expression analysis of *PagSAP11* under abiotic stress conditions (cold, heat and salt). The values represent the means ± standard deviations (SDs) of three biological replicates. Asterisks indicate significant differences by student *t*-test (^*^p < 0.05, ^**^p < 0.005). **(C)** Subcellular localization of *PagSAP11*-GFP protein. The green color indicates PagSAP11-GFP expression and red color means autofluorescence. Scale bar = 30 μm.

Thereafter, we determined the full-length coding sequence (CDS) of *PagSAP11*. The full-length CDS of *PagSAP11* without introns was 540 bp long, and encoded protein was estimated to comprise 179 amino acids, with one AN1 domain and one A20 domain (Figure 2A). The sequence data was submitted to GenBank (Accession number ON519817). The molecular weight of *PagSAP11* was predicted 19.65 kDa. We also performed a multiple alignment analysis based on the amino acid sequences of *PagSAP11* with genetically closed proteins from other plant species. The amino acid sequence of *PagSAP11* shared 98% identity with PtSAP11 and 84% with PtSAP4 from *P. trichocarpa,* 97% with PeSAP5 from *P. euphratica*, 67% with PpSAP5 from *P. persica*, 61% with AtSAP5 from *Arabidopsis*, 60% with VvSAP5 from *V. vinifera*, and 52% with OsSAP11 from *O. sativa* (Figure 2A). To identify the phylogenetic relationships of *PagSAP11*, we developed an unrooted phylogenetic tree using the protein sequence of *PagSAP11* and its orthologs (Figure 2B). As shown in Figure 2B, *PagSAP11* was clustered in the same clade as PtSAP11 and PeSAP5. In addition, *PagSAP11* was closely associated with AtSAP5, which is involved in conferring resistance towards salt stress, osmotic stress and water deficiency (Kang et al., 2011). These results suggest that *PagSAP11* is a suitable candidate gene associated with drought stress response.

**Figure 3:**
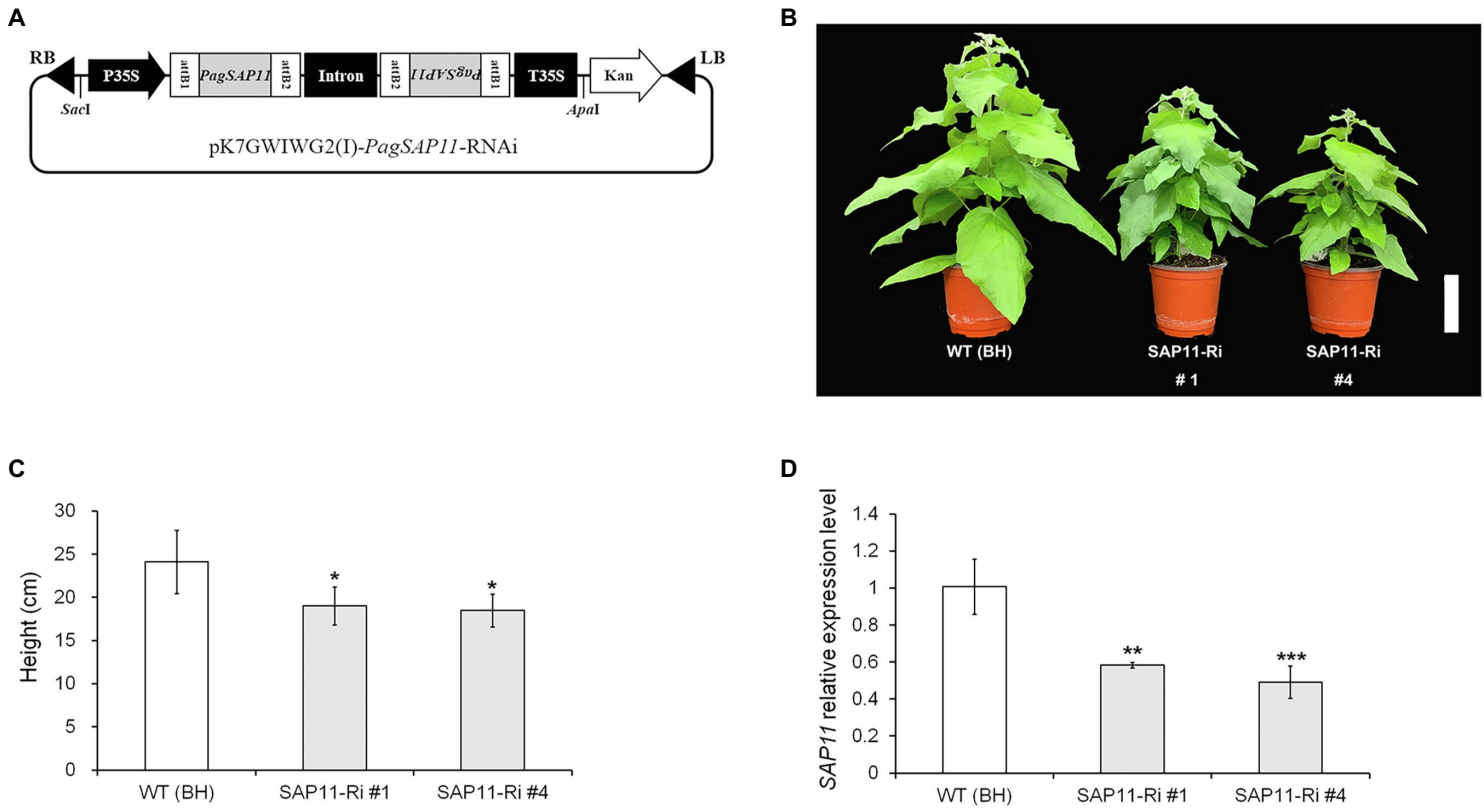
Generation of *PagSAP11*-RNAi transgenic poplar using *Agrobacterium*-mediated transformation. **(A)** Strategy for construction of expression vector pK7GWIWG2(I), containing the *PagSAP11-*RNAi. **(B)** Representative image of WT (clone BH) and independent two transgenic (SAP11-Ri #1 and #4) poplar plants grown for 2 months on soil after acclimation (*n* = 15). **(C)** Measurement of plant heights from **(B)**. **(D)** Relative *PagSAP11* transcript levels in each transgenic line. The expression levels of transgenic plants were confirmed using real-time qPCR. The values represent the means ± standard deviations (SDs) of three biological replicates. Asterisks indicate significant differences by student *t-*test (^*^p < 0.05, ^**^p < 0.005, ^***^p < 0.001). Scale bar = 10 cm.

To elucidate the molecular function of *PagSAP11*, we estimated expression patterns of *PagSAP11* gene in various tissues and under stress conditions. Transcript levels of *PagSAP11* were highest in young leaves (Figure 3A). Additionally, expression of *PagSAP11* increased under heat as well as drought stress conditions (Figure 3B; Supplementary Figure S1). Further, we analyzed the subcellular localization of *PagSAP11* by transient expression of *PagSAP11*-GFP fusion protein in protoplasts from hybrid poplars (Figure 3C). We observed strong GFP signals in the nucleus (Figure 3C), indicating that *PagSAP11* is a nuclear protein.

### Morphological Features of *PagSAP11* Transgenic Lines

To explore the function of *PagSAP11*, we generated both knockdown (SAP11-Ri) and overexpressing (SAP11-OX) transgenic poplars. Under normal growth conditions, the two independently transformed lines of SAP11-Ri (SAP11-Ri #1 and #4) exhibited reduced shoot height and developed lateral branches (Figures 1B,C). Transcript levels of *PagSAP11* were lower in knockdown transgenic poplars than in the WT poplar plants (Figure 1D). In contrast, *PagSAP11*-overexpressing plants also showed relatively reduced shoot height (Supplementary Figure S2) but exhibited a phenotype similar to that of WT plants. Overall, these results suggested that *PagSAP11* is associated with lateral shoot growth.

### Knockdown of *PagSAP11* Increases Drought Stress Tolerance

**Figure 4:**
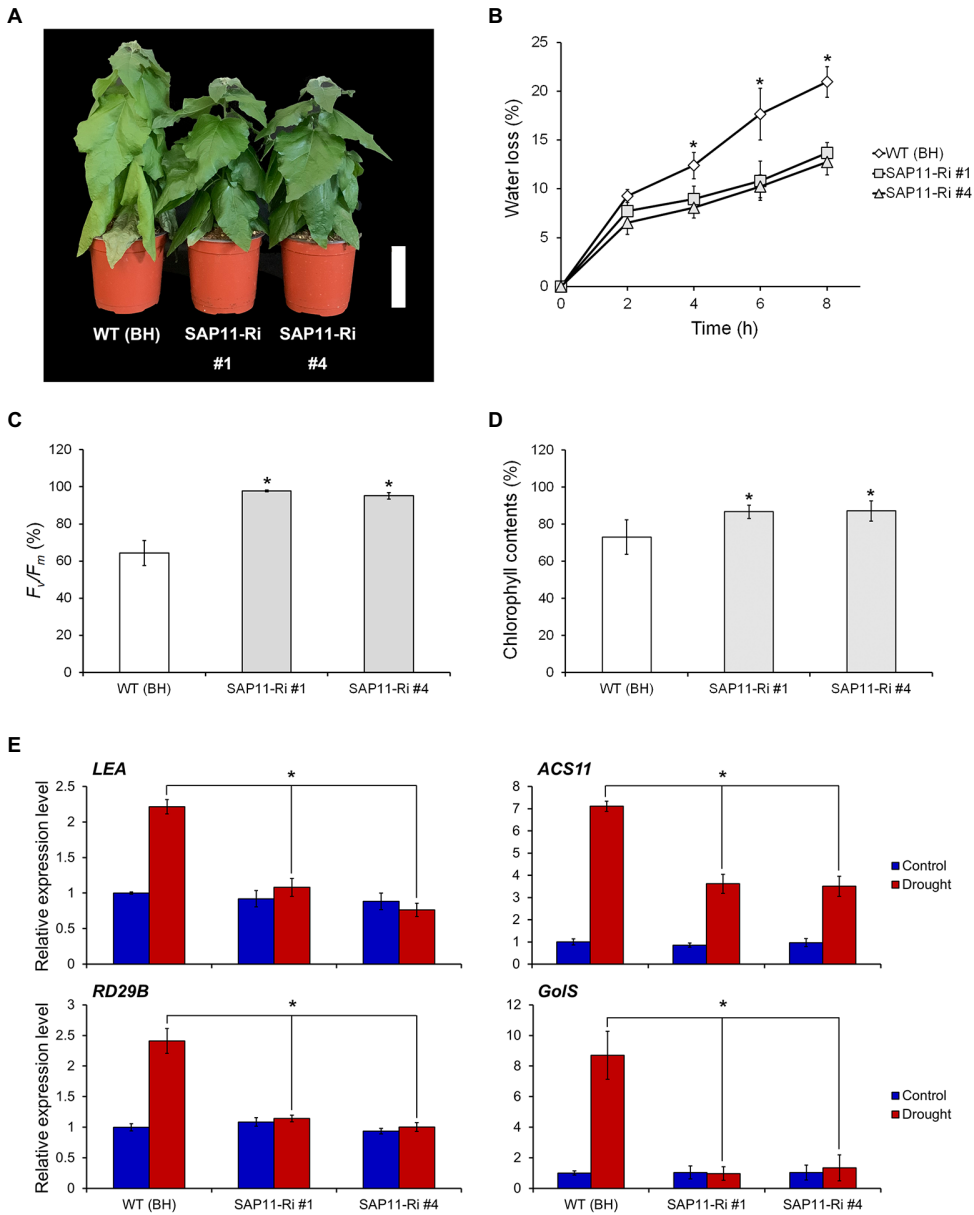
Knockdown of *PagSAP11* improves drought tolerance. **(A)** Phenotype of WT (BH clone) and transgenic poplars (SAP11-Ri #1, #4) after water withdrawal for 10 days. **(B-D)** Transpirational water loss **(B)**, photochemical efficiency **(C)**, and chlorophyll contents **(D)** were measured in detached leaves from WT and transgenic plants (*n* = 5). **(E)** Expression changes of drought-responsive genes in WT and transgenic poplars after water withdrawal for 3 days. The values represent the means ± standard deviations (SDs) of three biological replicates. Asterisks indicate significant differences by student *t*-test (^*^p < 0.05, ^**^p < 0.005). Scale bar = 10 cm.

To analyze the drought stress resistance of SAP11-Ri plants, we suspended the watering of 8-week-old WT and SAP11-Ri plants for 10 days. The plants were then kept in a growth room and maintained at low humidity. As a result, compared to the WT plants, SAP11-Ri plants showed drought stress tolerance even after 10 days without watering (Figure 4A). Thereafter, to evaluate the drought resistance of SAP11-Ri plants, we measured transpirational water loss of detached leaves from WT and transgenic plants. Leaves from SAP11-Ri plants had a significantly reduced water loss, while leaves from WT plants lost about 20% of their FW (Figure 4B). In addition, we also estimated photochemical efficiency of photosystem II (PSII) and chlorophyll contents in detached leaves from WT and SAP11-Ri plants. As shown in Figure 4C, the photochemical efficiency of PSII, a representative physiological marker of leaf senescence (Lee et al., 2016), rapidly declined in WT leaves compared to SAP11-Ri leaves. Consequently, chlorophyll contents of SAP11-Ri leaves were slowly decreased compared to WT leaves (Figure 4D). Moreover, we found that the expression levels of *LEA*, *RD29B* (*Responsive to Desiccation 29B*), *ACS11* (*1-Aminocyclopropane-1-Carnoxylate Synthase 11*) and *GolS* (*Inositol-3-α-Galactosyltransferase*) were increased in WT leaves, whereas they were slightly increased or unchanged in SAP11-Ri leaves (Figure 4E). In contrast, SAP11-OX lines showed a drought-sensitive phenotype under drought stress (Supplementary Figure S3). Taken together, these results indicate that the knockdown of *PagSAP11* confers drought resistance.

### Down-Regulation of *PagSAP11* Affects Lateral Bud Outgrowth

**Figure 5:**
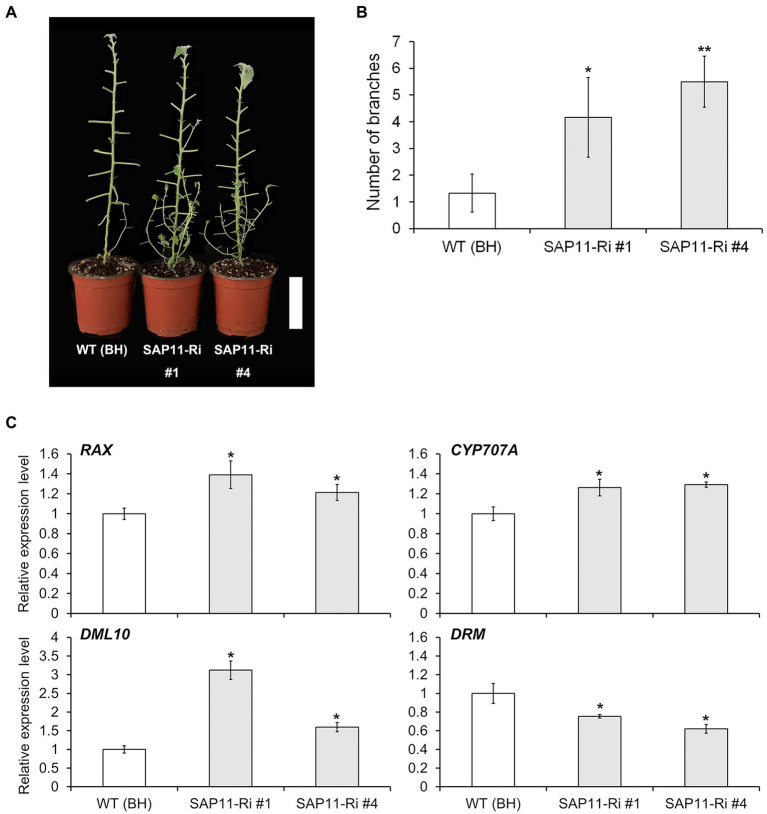
*PagSAP11*-RNAi hybrid poplars exhibit increased sylleptic branching. **(A)** Sylleptic branching in representative defoliated WT (clone BH) and transgenic poplars. **(B)** Sylleptic branch number in WT and transgenic poplars grown for 2 months on the soil after acclimation (*n* = 5). **(C)** Relative expression levels of genes involved in bud outgrowth. The values represent the means ± standard deviations (SDs) of three biological replicates. Asterisks indicate significant differences by student *t*-test (^*^p < 0.05, ^**^p < 0.005). Scale bar = 10 cm.

As described above, SAP11-Ri plants showed reduced shoot height and an increased number of lateral branches compared to WT plants (Figure 1). To estimate association between *PagSAP11* and shoot branching, we measured the number of lateral branches in WT and SAP11-Ri plants. As a result, SAP11-Ri plants showed increased branch numbers compared with WT plants (Figures 5A,B), whereas the SAP11-OX plants produced only a few branches (Supplementary Figures S4A,B). Next, we analyzed the expression levels of genes involved in axillary bud formation and bud dormancy. The mRNA abundances of *DML10* (*DEMETER-like demethylase*), *CYP707A* (*Cryptochrome P450 707A family*) and *RAX* (*Regulator of Axillary meristems*) were increased in SAP11-Ri plants, while the transcript levels of *DRM* (*Dormancy-associated protein 1*) were reduced (Figure 5C). In addition, the transcript levels of these genes were unchanged or decreased in SAP11-OX plants compared with WT plants (Supplementary Figure S4C). However, the expression levels of auxin, cytokinin and strigolactone-related genes did not change significantly between SAP11-Ri and SAP11-OX plants compared with WT plants (Supplementary Figure S5). Overall, these results indicate that *PagSAP11* is involved in shoot branching *via* regulating axillary bud formation and bud dormancy.

### LMO Field-Grown Transgenic Hybrid Poplar Trees Also Showed Increased Lateral Shoot Branching

**Figure 6:**
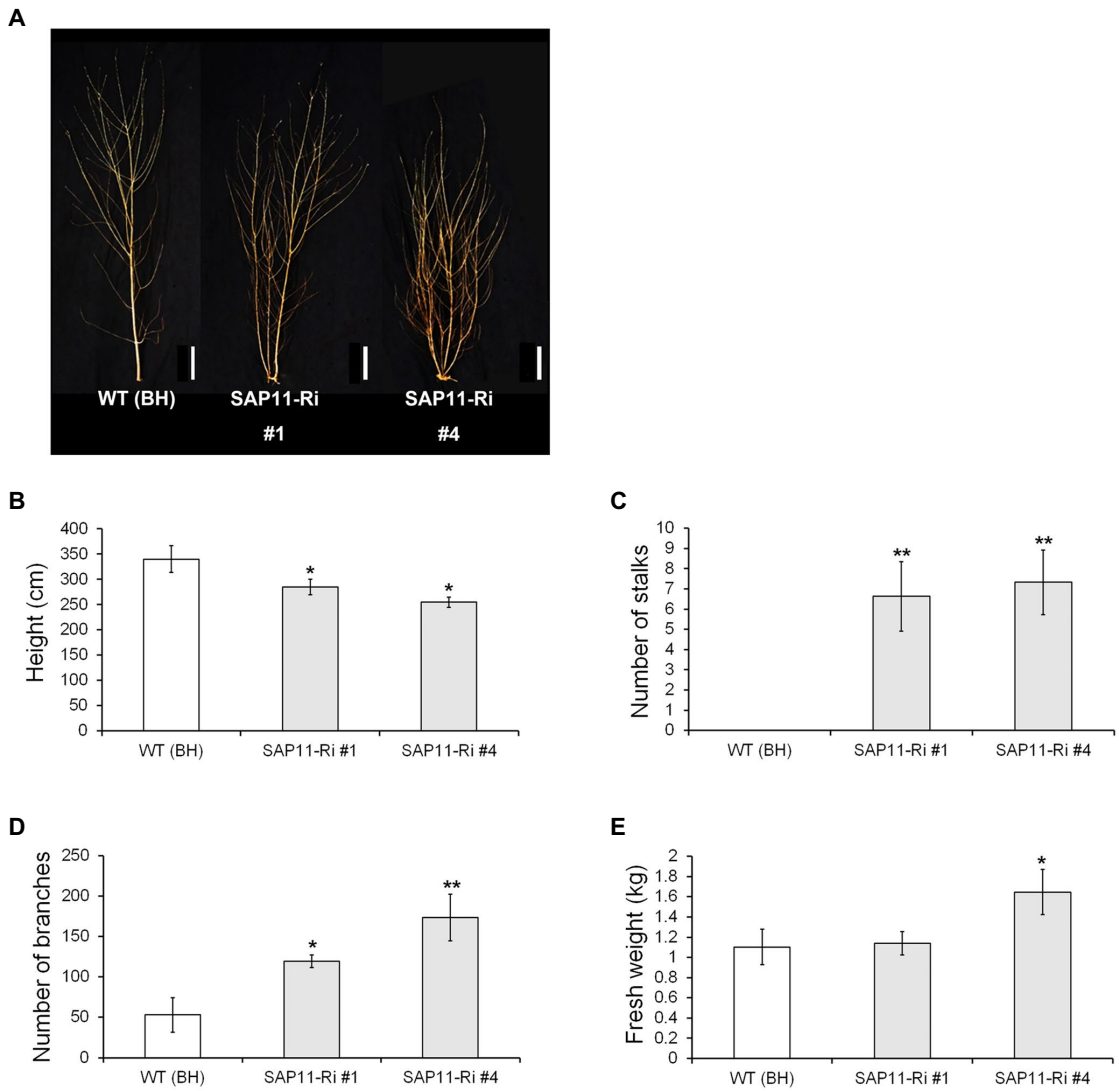
Growth-related data recorded from *SAP11*-engineered poplars of the field trial during second cultivation cycle. **(A)** Image of representative WT (clone BH) and transgenic poplar grown in the LMO field. (**B-D**) Shoot height **(B)**, number of side stalks **(C)** and number of sylleptic branches **(D)** in WT and transgenic poplars (*n* = 10). **(E)** Aboveground biomass yields of the WT and *SAP11*-engineered poplars. The values represent the means ± standard deviations (SDs) of three biological replicates. Asterisks indicate significant differences by student *t*-test (^*^p < 0.05, ^**^p < 0.005), Scale bar = 30 cm.

To evaluate the enhanced lateral bud outgrowth of SAP11-Ri plants under LMO field conditions, we planted SAP11-Ri transgenic and WT poplars in the LMO facility and monitored their growth performance. After 3 months of active growth in the spring to summer seasons of the second year, we measured stem height and diameter of transgenic poplars (Figure 6). Selected trees were photographed in the winter (Figure 6A). SAP11-Ri poplars exhibited decreased shoot height compared to the WT poplars (Figure 6B). Next, we estimated the number of their side stalks and sylleptic branches. As a result, SAP11-Ri poplars showed increased number of side stalks and sylleptic branches (Figures 6C,D). Moreover, SAP11-Ri poplars showed increased dry weight due of an increase in lateral shoot branching (Figure 6E). Therefore, these results suggest that increased sylleptic shoot branching supports maintenance of biomass in SAP11-Ri plants.

## Discussion

Drought stress severely affects plant growth and productivity so that adjusting plant architecture in response to drought stress is important for plant survival (El-Sappah et al., 2021). Lateral shoot branching in plants determines plant architecture, which is complicatedly regulated by genetic factors, phytohormones, nutrition, and the environmental signals. Stress-associated protein (SAP) genes have been reported that are involved in response to abiotic stress in various plant species, including woody plants. In this study, we identified that expression of *PagSAP11* was induced under drought stress (Supplementary Figure S1). We used a transgenic approach to silence and upregulate the expression of *PagSAP11* to investigate its function. As a result, we explored that knockdown of *PagSAP11* induced tolerance to drought stress, whereas overexpressing lines exhibited a drought-sensitive phenotype (Figure 4; Supplementary Figure S3). Hence, we concluded that *PagSAP11* provides an important adaptation strategy under drought stress conditions by modulating plant architecture.

Poplar species and their hybrids are considered as important industrial stemwood due to their rapid growth and high productivity (Larchevêque et al., 2011). Among these, hybrid poplar (*P. alba* × *P. tremula* var. *glandulosa*) can tolerate flooding, has extensive root system, and has salt and cold tolerance (Qiu et al., 2019). Additionally, hybrid poplar has been used in many researches due to their high rates of transformation and well-documented genomic resources (Bae et al., 2021).

Amino acid sequence of *PagSAP11* shared 98% similarity with that of PtSAP11 (Figure 2). Jin et al. reported that *PtSAP11* has one C-repeat (CRT)/dehydration responsive elements (DRE) element in its upstream of the translation start site. A DRE with a 9 bp conserved core sequence (5’-TACCGACAT-3′) was identified in the promoter of the drought-responsive gene *rd29A* in *Arabidopsis* (Yamaguchi-Shinozaki and Shinozaki, 1993). CRT with the 5 bp core sequence (5’-CCGAC-3′) was identified in the promoter of cold-inducible genes from *Arabidopsis* (Baker et al., 1994). DREB proteins have been reported that are important transcription factors which regulate stress signal transduction and modulate gene expression by interacting with a DRE/CRT *cis*-element present in the promoter region of various abiotic stress-responsive genes (Lata and Prasad, 2011). *PagSAP11* also is likely to have a DRE element in its promoter region, suggesting that DREBs can bind to the promoter region of *PagSAP11* to induce expression of *PagSAP11* in response to drought stress. Structural analysis revealed that *PagSAP11* consists of one AN1 domain and one A20 domain (Figure 2). Although the biochemical function of these domains is still largely unknown, OsSAP8 and PagSAP1 are localized in the cytoplasm and presumably function *via* protein–protein interactions mediated by their A20 and AN1 zinc finger domains (Kanneganti and Gupta, 2008; Yoon et al., 2018). In addition, it was identified that the OsSAP1 could interact with self, its close homolog OsSAP11 and a rice receptor-like cytoplasmic kinase, OsRLCK253 *via* A20 domain (Giri et al., 2011). AtSAP5, the ortholog of *PagSAP11* in *Arabidopsis*, exhibits E3 ubiquitin ligase activity *in vitro* (Kang et al., 2011). Moreover, it is generally known that many of the zinc-finger domain proteins act as transcriptional regulators. *PagSAP11* protein was localized in the nucleus (Figure 3C), indicating that *PagSAP11* may act as a transcriptional regulator in the nucleus.

Branching or branch number is regarded as an integral trait of tree crown architecture. Since this trait determines leaf area index (LAI), it contributes directly to the biomass production in the short-rotation poplar coppice (Ding et al., 2021). Shoot branching is an orchestrated process involved in a wide range of external environmental, internal, physiological and developmental factors (Leyser, 2009). Previous studies have reported that phytohormone such as auxin, cytokinin and strigolactone are involved in lateral shoot branching as internal factors (Cline and Dong-Il, 2002; Muhr et al., 2016; Barbier et al., 2019; Yang et al., 2020). The role of auxin in shoot branching has been well established through studies in which the removal of the shoot tip of plants stimulates axillary bud outgrowth and this growth could be suppressed by applying the plant hormone auxin to the stump of decapitated plants (Thimann and Skoog, 1933). Moreover, it was recently identified that the apically derived auxin transported in the stem upregulates expression of strigolactone synthesis genes and downregulates cytokinin levels, resulting in the inhibition and promotion of bud outgrowth, respectively (Muhr et al., 2016; Barbier et al., 2019). However, in this study, the expressions of auxin-, cytokinin- and strigolactone-related genes were similar to WT, SAP11-Ri and SAP11-OX plants (Supplementary Figure S5). In contrast, the transcript levels of *RAX1*, which encodes an MYB transcription factor involved the initiation of axillary meristems (Jia et al., 2020) were increased in SAP11-Ri. Furthermore, the expression levels of *DML10*, a gene involved in bud break (Conde et al., 2019), and *CYP707A*, a gene related to bud dormancy (Zhao et al., 2021), were also upregulated in SAP11-Ri. However, the mRNA abundance of *DRM*, a marker of bud dormancy (Yoon et al., 2017), was decreased in SAP11-Ri compared with the WT plants (Figure 5). On the other hands, knockdown and overexpressing of *PagSAP11* resulted in reduced plant height, suggesting that the phenotypes finely tuned and influenced by various factors. For example, in rice, overexpression and knockout of *OsCKX9* resulted in retarded shoot growth (Duan et al., 2019). These findings indicate that *PagSAP11* regulates bud dormancy different from hormone-regulated pathway.

In this study, we investigated the gene characteristics and functions of *PagSAP11* in drought stress response and shoot branching in hybrid poplar. Our results suggest that *PagSAP11* negatively regulates response to drought stress and the growth of lateral branches. Downregulation or overexpression of *PagSAP11* can increase or decrease drought stress resistance in transgenic plants, respectively. Moreover, reduced expression of *PagSAP11* can induce lateral shoot branching in the transgenic poplars, in greenhouses as well as in the LMO field. Overall, our research suggests that *PagSAP11* is a potential candidate gene to engineer woody plants that maintain their biomass under drought stress conditions.

## Data Availability Statement

The data presented in the study are deposited in the GenBank, accession number ON519817.

## Author Contributions

SP, HC, S-KY, and H-AJ performed the experiments, analyzed the data, and wrote and revised the manuscript. EK-B, HC, Y-IC and HL designed the study and wrote and edited the manuscript. All authors contributed to the article and approved the submitted version.

## Funding

This study was supported in part by the National Institute of Forest Science (project no. FG0702-2018).

## Conflict of Interest

The authors declare that the research was conducted in the absence of any commercial or financial relationships that could be construed as a potential conflict of interest.

## Publisher’s Note

All claims expressed in this article are solely those of the authors and do not necessarily represent those of their affiliated organizations, or those of the publisher, the editors and the reviewers. Any product that may be evaluated in this article, or claim that may be made by its manufacturer, is not guaranteed or endorsed by the publisher.

## Footnotes

^1^ http://fokker.wi.mit.edu

^2^ http://phytozome-next.jgj.doe.gov
