# Supplementary material for: Knockdown of PagSAP11 Confers Drought Resistance and Promotes Lateral Shoot Growth in Hybrid Poplar (Populus alba × Populus tremula var. glandulosa)
Source: Front Plant Sci. 2022 Jun 24;13:925744. doi: 10.3389/fpls.2022.925744 (PMC9263715; doi:10.3389/fpls.2022.925744)
Supplement: Supplementary file 1 [file Data_Sheet_1.PDF]

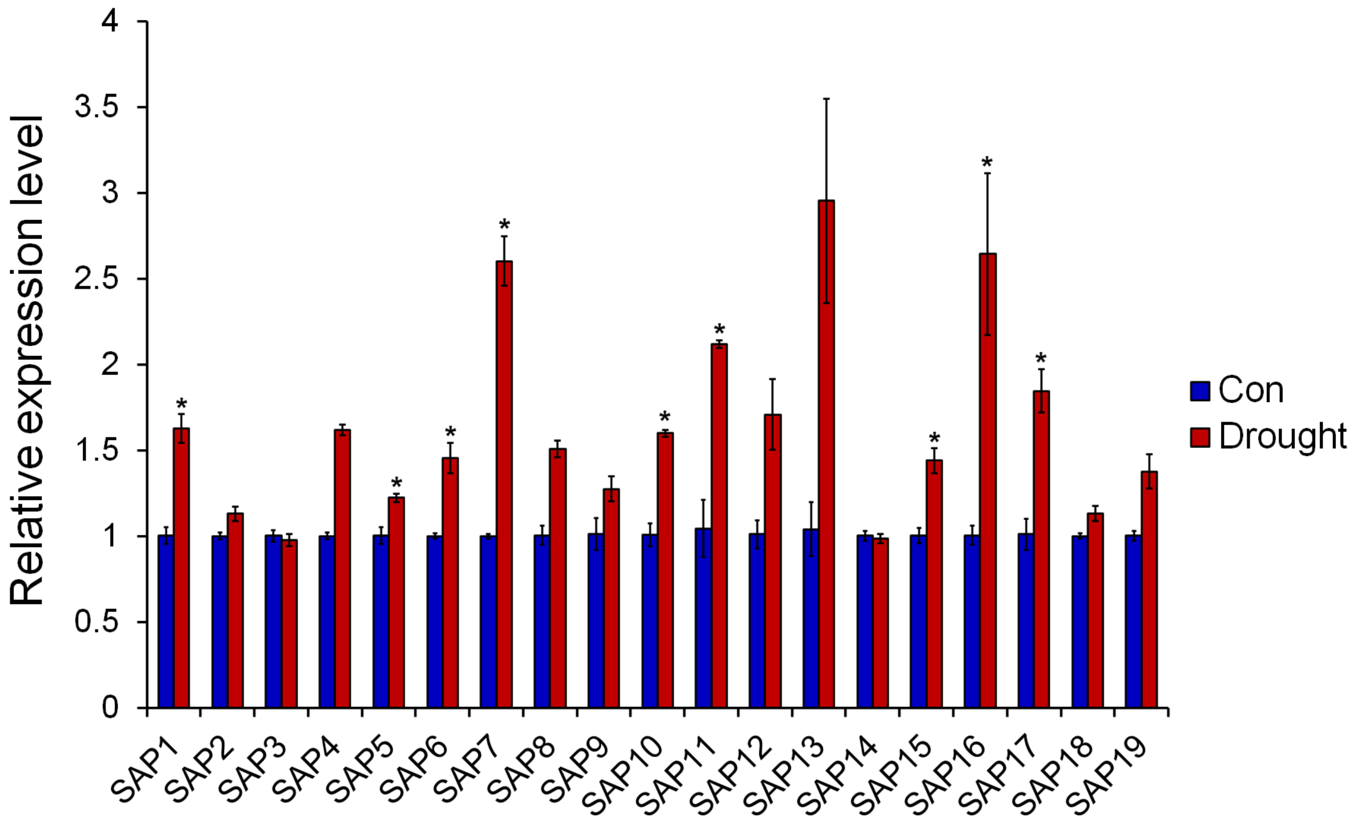

Supplementary Figure 1. Expression of stress-associated protein (SAP) genes under drought stress in hybrid poplar. Asterisks (*) indicates *P*<0.05.

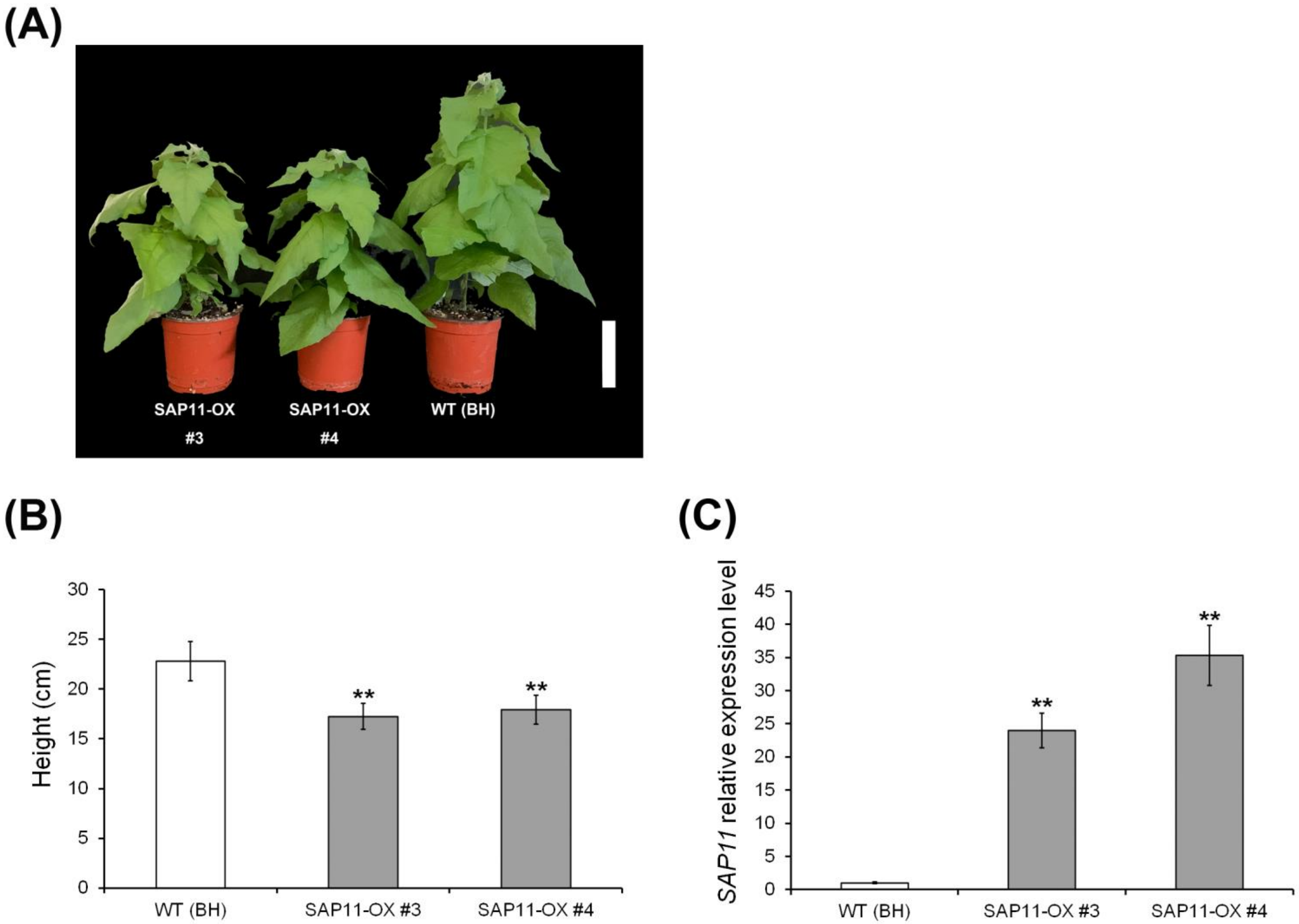

Supplementary Figure 2. Phenotype of *PagSAP11*-overexpressing lines.

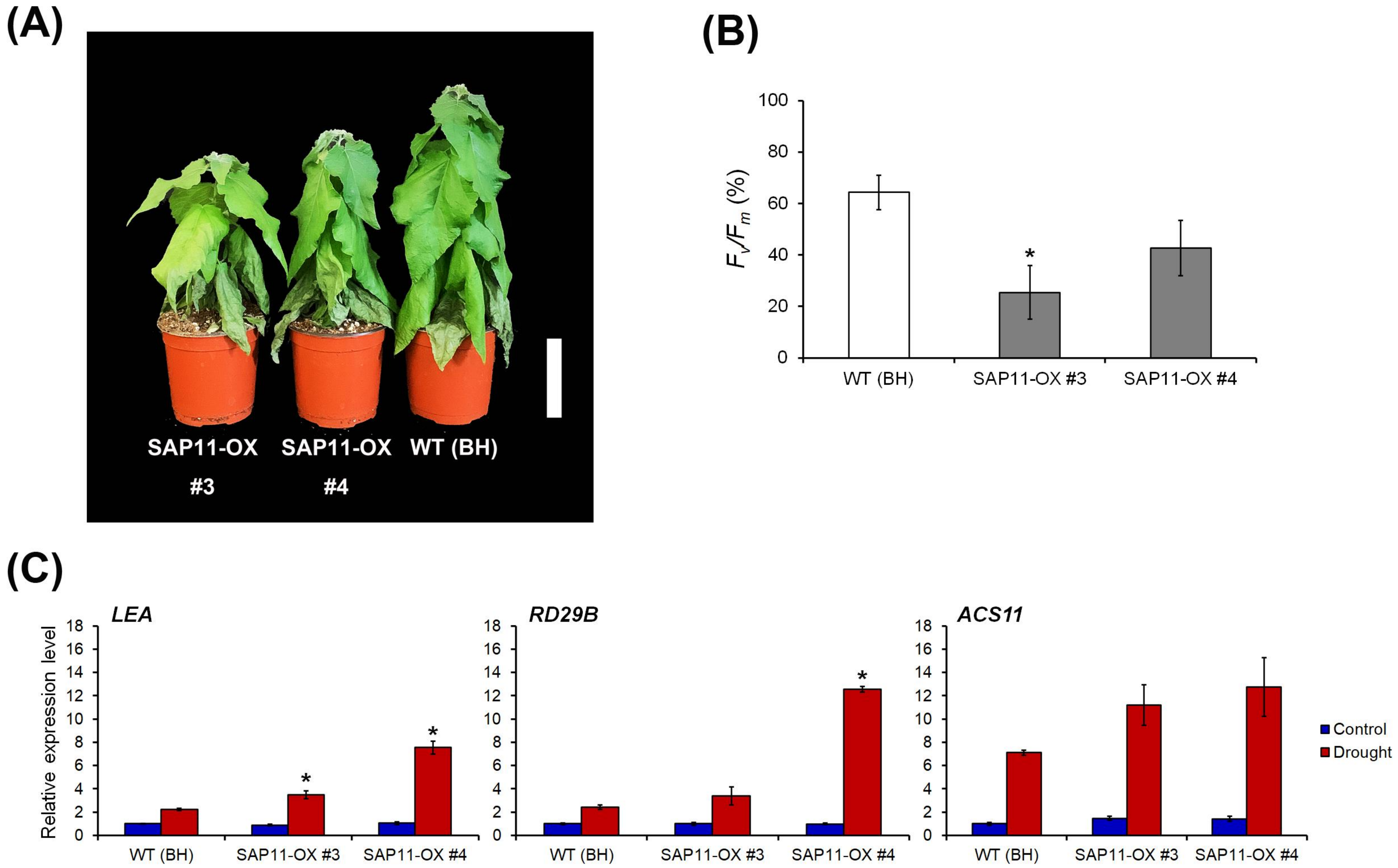

Supplementary Figure 3. *PagSAP11*-OX plants showed sensitive phenotype in dehydration.

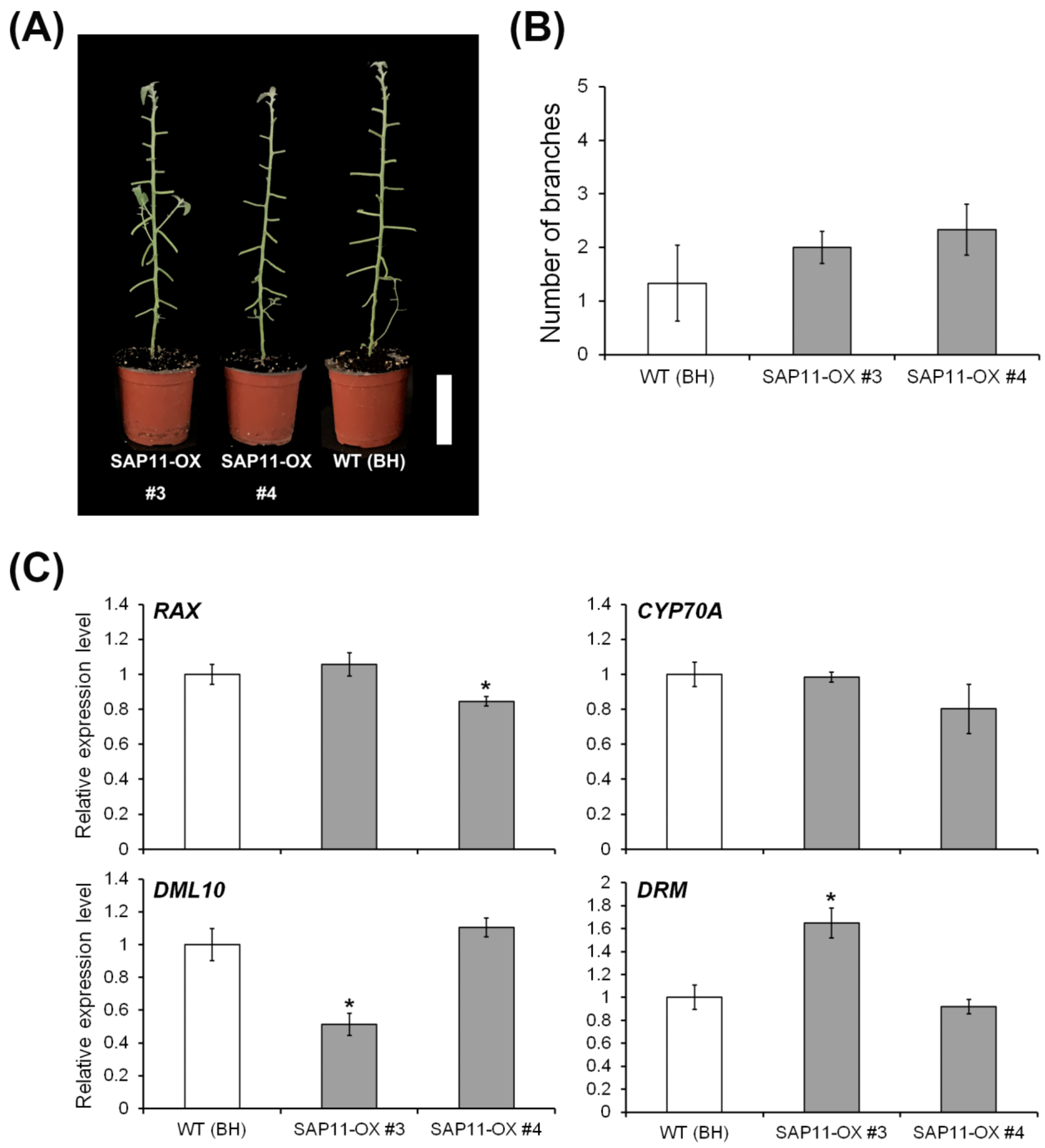

Supplementary Figure 4. Number of lateral branches of *SAP11*-OX plants not significantly changed in comparison with WT plants.

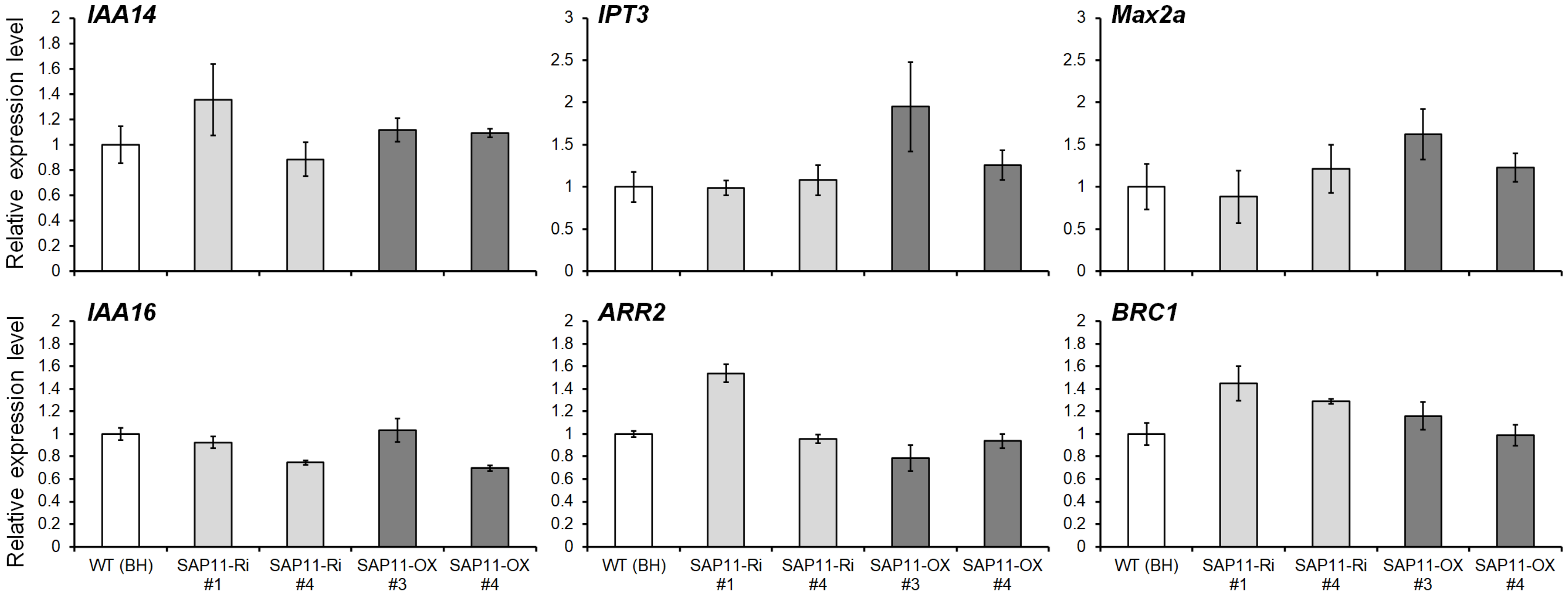

Supplementary Figure 5. Expression levels of phytohormone-related genes in WT, *SAP11*-Ri and *SAP11*-OX plants.
